# Right Upper Lobe Partial Anomalous Pulmonary Venous Connection

**DOI:** 10.1155/2014/249896

**Published:** 2014-02-16

**Authors:** Christos Tourmousoglou, Christina Kalogeropoulou, Efstratios Koletsis, Nikolaos Charoulis, Christos Prokakis, Panagiotis Alexopoulos, Emmanoil Margaritis, Dimitrios Dougenis

**Affiliations:** ^1^Cardiothoracic Department, University Hospital of Patra, 26504 Rio, Patra, Greece; ^2^Department of Radiology, University Hospital of Patra, 26504 Rio, Patra, Greece

## Abstract

Partial anomalous pulmonary venous return (PAPVR) is a left-to-right shunt where one or more, but not all, pulmonary veins drain into a systemic vein or the right atrium. We report a case of a 45-year-old male with PAPVR to superior vena cava which was incidentally discovered during a right lower bilobectomy for lung cancer.

## 1. Introduction 

Partial anomalous pulmonary venous connection (PAPVC) is an uncommon congenital anomaly in which one or more pulmonary veins drain into a systemic vein or the right atrium rather than the left atrium. Anomalous right-sided pulmonary veins might drain into the superior vena cava, inferior vena cava, right atrium, azygos vein, portal vein, or hepatic vein. Besides, anomalous left-sided pulmonary veins could drain into the left brachiocephalic vein, coronary sinus, or hemiazygos vein. The PAPVC might occur as an isolated anomaly or might be combined with atrial septal defect (ASD) [1]. The PAPVC to the superior vena cava occurs in about 10–15% of all patients with ASD.

We report a case of a 45-year-old male with PAPVC that was incidentally discovered during a right lower bilobectomy for lung cancer.

## 2. Case Report

**Figure 1:**
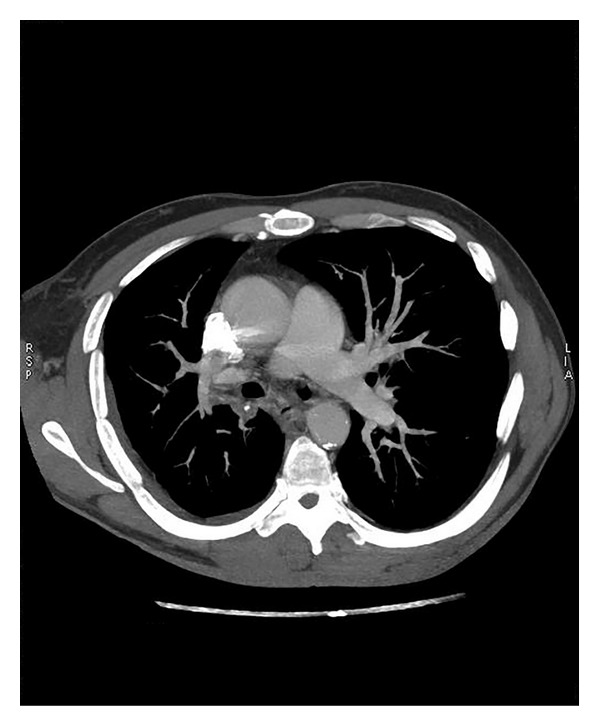
Multidetector computed tomography showed a PAPVC from the right upper lobe to the superior vena cava (arrow).

**Figure 2:**
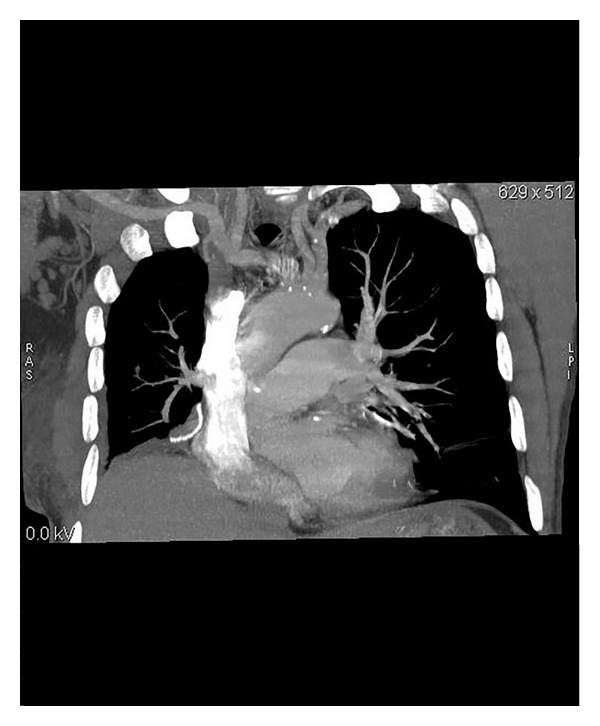
Another coronal oblique CT image denoted a PAPVC from the right upper lobe to the superior vena cava (arrow).

A 45-year-old male had a persistent cough for two months and his chest X-ray showed a nodule in the right lower lobe. A chest computed tomography revealed a mass (5.8 × 6.6 × 6.3 cm) in the right lower lobe that was attached to the right hilum and around the bronchus. Bronchoscopy showed a lesion at the entrance of the right lower lobe that obstructed 30% of the bronchus and pathological examination of a biopsy revealed adenocarcinoma. Spirometry revealed the following values of FVC: 3.17 L (60%) and FEV_1_: 2.79 L (77%), while electrocardiography showed normal cardiovascular activity. Clinical examination did not reveal marked abnormalities or evidence of vascular shunt. Surgery was performed through a left posterolateral thoracotomy under one-lung ventilation. An anomalous pulmonary vein was incidentally discovered and the right upper lobe vein drained into the superior vena cava. A right bilobectomy was performed. The right upper lobe with the anomalous variation remained as it was. The patient had an uneventful postoperative course. After the operation, a careful examination and reconstruction of the CT images revealed the anomaly (Figures 1 and 2).

## 3. Discussion

Partial anomalous pulmonary venous connection is a relatively uncommon congenital anomaly that is found in only 0.5–0.7% of the general population [2, 3]. Asymptomatic PAPVC without an ASD is extremely rare. The greatest number of cases of PAPVC is located in the right lung and the anomalous vein or veins are often connected to the right atrium or the superior vena cava [4].

All PAPVC are left-to-right shunts but more than 50% of the pulmonary flow drain into the right side of the heart and so clinical manifestations such as fatigue, dyspnea, syncope, atrial arrhythmias, right heart failure, and pulmonary hypertension might occur rarely [5, 6]. Generally, PAPVC is symptomatic and it is often associated with other congenital heart defects, especially ASD, reportedly in 80–90% of the cases [7, 8]. No associated risk factors have been identified for its development.

Embryonic development of the pulmonary veins occurs early in development of the cardiovascular system. The main theory is that the initial drainage is via the splanchnic plexus into the cardinal and umbilical vitelline. A craniocaudal outpouching forms in the sinoatrial region of the heart with extension to lung buds. As caudal regression occurs, the cranial portion develops into the common pulmonary vein, which is incorporated into the left atrial wall. Partial anomalous pulmonary venous return occurs due to failure of connection between the common pulmonary vein and the splanchnic plexus [9, 10].

PAPVC is usually diagnosed by transthoracic echocardiography (TTE), transesophageal echocardiography (TEE), or catheter angiography. The information provided by echocardiography is sometimes not sufficient. Besides, pulmonary angiography by right heart catheterization might not reveal details about the anatomy of small accessory and anomalous vessels [11]. Another examination such as 128-slice multidetector computed tomography (MDCT) scan is helpful in defining ASDs and PAPVR. ECG-gated MDCT offers the possibility of a noninvasive and rapid acquisition with high resolution. All of the anomalous veins and associated cardiovascular defects are observed by MDCT-angiography [12]. The isotropic voxel size and spatial resolution offer the possibility to discover small vessels and shunts with multidimensional reconstructions with the usage of advanced workstations [13]. But it is really a concern about the radiation dose of MDCT especially in young patients [14]. The usage of ECG attenuation techniques limits the exposure during the less informative parts of the cardiac cycle. The success of this technique depends on the usage of premedication, ECG-gating, and special technical protocols. Data processing of multidimensional images might be time consuming but 2D and 3D images are valuable in planning the operation [13].

MRI will also demonstrate the abnormal pulmonary venous connection, but it could better depict an associated ASD. Chest radiography is often normal and secondary signs of a left-to-right shunt such as cardiomegaly, pulmonary vascular prominence, and pulmonary artery hypertension could be seen [8].

If the lung cancer is located in the same lobe as the PAPVC, the patient could have either a lobectomy or an ipsilateral pneumonectomy with an uneventful postoperative course. But if the PAPVC is located in a different lobe, a lobectomy or a contralateral pneumonectomy might result in heart failure [3, 15]. In case that major pulmonary resections are performed for patients with PAPVC, it is critical to correct this anomaly for preventing heart failure.

There are a number of operative procedures for the correction of PAPVR. An anomalous vein on the left side is implanted directly into the left auricular appendage or the left atrium. A number of postoperative complications such as kinking, stenosis of the corrected pulmonary vein, obstruction, and arrhythmias are correlated with these procedures [16, 17]. Surgical repair for PAPVC to SVC consists of complete closure of the septal defect, with redirection of the anomalous pulmonary veins into the left atrium without SVC obstruction, pulmonary venous obstruction, or injury of the sinus node or its blood supply. In case that the veins enter the SVC in an upright position, the surgical treatment is more demanding.

Warden et al. reported a technique for the correction of high PAPVC in which the SVC was divided above the orifices of the anomalous pulmonary veins and the cephalic end of the divided SVC was anastomosed directly to the right atrial appendage. A patch was inserted into the right atrium and diverted the pulmonary blood flow from the orifice of the SVC through the sinus venosus ASD. Then, the caudal end of the divided SVC was closed by sutures. This technique has the advantage of decreased manipulation of the cavoatrial junction while avoiding the creation of conduits inside the SVC [18].

In conclusion, PAPVC is an uncommon congenital anomaly and is incidentally found during an operation for lung cancer. Coronal, sagittal, and 3D volume rendered reformatted CT images could help in the diagnosis of this entity. The best surgical approach for lung resection in patients with PAPVC should be considered carefully for preventing postoperative heart failure.
